# Radial shape discrimination testing for new-onset neovascular age-related macular degeneration in at-risk eyes

**DOI:** 10.1371/journal.pone.0207342

**Published:** 2018-11-08

**Authors:** Noelia Pitrelli Vazquez, Simon P. Harding, Heinrich Heimann, Gabriela Czanner, Paul C. Knox

**Affiliations:** 1 Department of Eye and Vision Science, University of Liverpool, Liverpool, United Kingdom; 2 St Pauls Eye Unit, Royal Liverpool University Hospital, Liverpool, United Kingdom; 3 Department of Applied Mathematics, Liverpool John Moores University, Liverpool, United Kingdom; Eye Hospital, Charité, GERMANY

## Abstract

We investigated the performance of the handheld radial shape discrimination (hRSD) test in detecting the development of neovascular AMD (nAMD) in a prospective, longitudinal, observational study. Patients diagnosed with unilateral nAMD, with no nAMD in the other eye (the study eye, SE), completed the hRSD test on consecutive, routine clinic visits up to a maximum of 12, or until they were diagnosed with nAMD in the SE based on slit-lamp biomicroscopy and spectral-domain OCT assessment, with fluorescein angiography confirmation. Masked grading was carried out to confirm the diagnosis of nAMD, and to ensure no cases of nAMD were missed. Receiver operating characteristics (ROC) analysis was used to explore the diagnostic performance of the hRSD test relative to clinical diagnosis. Data were available from 179 patients of whom 19 (10.6%; “converters”) developed nAMD in the SE. The mean hRSD threshold at conversion was -0.47 (95% CI -0.38 to -0.55) logMAR compared to -0.53 (-0.50 to -0.57) logMAR in 160 non-converters. hRSD threshold in the converters began to decline 190 days before diagnosis of nAMD. The ROC curve demonstrated that at an hRSD cut-off of -0.60 logMAR, sensitivity was 0.79 (0.54–0.94) with a specificity of 0.54 (0.46–0.62); positive and negative predictive values were 0.16 and 0.96 respectively. We conclude that the hRSD test has moderate sensitivity for detecting the earliest stages of nAMD in the at-risk fellow eyes of patients with unilateral nAMD, compared to clinical diagnosis. Given its relative inexpensiveness, ease of use and the inherent connectivity of the platforms it can be presented on, it may have a role in early detection of nAMD in the population at large.

## Introduction

Age-related macular degeneration (AMD) is the leading cause of vision loss in developed countries, and with the ageing of the population its prevalence is expected to increase[1]. However, the prognosis for patients with neovascular AMD (nAMD) has considerably improved with the introduction of intravitreal therapy against vascular endothelial growth factor (VEGF). Pivotal clinical trials demonstrated that anti-VEGF treatment improves visual acuity (VA) after twelve months of monthly treatment, gains that are maintained at 24 months (see Solomon, 2014, for review[2]). Longer term follow-up studies have confirmed that treatment leads to a markedly improved outcome compared to the devastating visual decline that results from untreated nAMD[3, 4]. However, in the real world of rising patient numbers and economic and service delivery challenges, outcomes have not been as positive as in the clinical trials[5].

Early detection and diagnosis of nAMD, leading to earlier treatment, results in better visual outcomes[6–10]. However, many patients only seek treatment when they become perceptually aware of changes in their vision. Recent analysis of trial and other data has demonstrated significant visual loss prior to diagnosis[11]. Screening, self-testing or home monitoring (perhaps using some form of tele-monitoring[12]) might facilitate earlier detection, but require appropriate tests. Diagnostic testing currently relies on fundus fluorescein angiography (FFA), increasingly supplemented by optical coherence tomography (OCT)[13]. These clinic-based approaches are relatively expensive, can be time consuming (and in the case of FFA invasive) and none can be self-administered.

**Fig 1:**
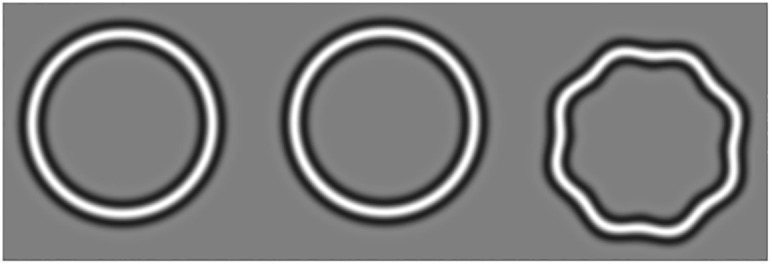
Example of the stimuli used in the handheld radial shape discrimination (hRSD) test[17]. The test employed three radial frequency patterns, presented on an Apple iPod Touch. The vertical position of each pattern was randomly varied, as was the position of the target (distorted) pattern (the pattern on the right in this example).

MyVisionTrack (mVT, Vital Art and Science Inc, Dallas, USA) is an application that has FDA approval for use in monitoring of macular disease, and runs on a range of mobile devices. The test is based on shape perception and exploits the sensitivity of the human visual system to distortions in circular radial frequency patterns, in this case circular contours with a cross-sectional luminance profile defined by a radial fourth derivative of a Gaussian [14] (see Fig 1 for example stimuli). As developed by Wang and colleagues [15–17], these radial frequency patterns are deformed by applying a sinusoidal modulation to the radius at a fixed frequency (8 cycles/360°) around the circumference. When embedded in a psychophysical staircase procedure, this provides a means of determining the minimal radial modulation amplitude necessary to make it possible to distinguish a distorted radial frequency pattern from a perfect one, defining a radial shape discrimination (RSD) threshold. Because of the sensitivity of the human visual system to these distortions, RSD thresholds fall into the hyperacuity range. While RSD threshold reaches maturity later than resolution acuity, once adult thresholds are reached they remain stable in the absence of pathology, and are relatively resistant to normal healthy ageing compared to VA[16, 18]. The development of AMD has been demonstrated to increase RSD thresholds [15] and test performance has been shown to be related to the severity of macular disease[17]. Despite the widespread deployment of this test, and its commercial availability (in the form of the mVT application in the US), prospectively collected diagnostic performance data is not yet available.

Our objective therefore was to investigate the diagnostic performance of the RSD test, presented on a small mobile device (the handheld RSD test, hRSD; the index test), for the detection of nAMD using the clinical diagnosis of nAMD, confirmed by FFA, as the reference standard. We followed patients who were receiving treatment for nAMD in one eye, whose other eye (the study eye, SE) was confirmed to have no nAMD when they entered the study. Because these eyes are considered at high risk of developing nAMD, they are routinely monitored[19–21] particularly as development of disease in the second eye has a major impact on the patient’s quality of life. We anticipated that this study design would allow us to capture test performance around the time of the development of nAMD in a proportion of study eyes[22] and provide summary data for test performance in high risk eyes that did and did not develop nAMD and eyes with diagnosed nAMD at the initiation of treatment. It would also provide comparative data with alternative tests examined using the same study design[22].

## Methods

### Participants

We conducted a prospective, longitudinal, observational, single centre study that complied with the tenets of the Declaration of Helsinki and was approved by an appropriate UK Health Research Authority/Research Ethics Committee (North West—Preston Research Ethics Committee; 13/NW/0449). Written, informed consent was obtained from all participants prior to any study procedures. We recruited patients attending a UK National Health Service clinic for the assessment and treatment of nAMD in their first eye (FE), whose other eye (the study eye, SE) was clear of nAMD, with no other sight threatening macular pathology. Patients were identified and approached based on clinical records and previous OCT scans (where available) between September 2013 and December 2015. Inclusion and exclusion criteria are listed in Table 1. In reporting this study we have followed the STARD 2015 guidelines[23].

**Table 1:** Study inclusion and exclusion criteria.

Inclusion Criteria	Exclusion Criteria
Over 50 years of age Willing and able to give informed consent for participation Ability to understand and perform the study tests (hRSD test and VA) Diagnosis of nAMD in the first eye (FE) with no nAMD in the study eye (SE)	Diabetes SE VA worse than 0.4 logMAR Other sight threatening conditions affecting the macula of the SE, including GA, ERM, VMT, laser scars.

SE: Study eye; FE: Eye diagnosed with nAMD; GA: geographic atrophy, ERM: epiretinal membrane, VMT: vitreomacular traction

### Procedures

Patients’ study visits were the same day as their standard clinical appointments for assessment and treatment of their FE, approximately every 4–12 weeks depending on the anti-VEGF drug they were receiving and the stability of their condition. We collected hRSD test data along with data from tests performed as part of routine care which included measurement of VA (using EDTRS charts) and OCT.

The hRSD test was performed using the handheld, spatial, three alternative forced choice (3AFC) version of the test presented on an Apple iPod touch (see Wang et al, 2013, for details of the test[17]). Briefly, three radial frequency patterns were presented on the iPod screen, one of which was distorted by sinusoidal modulation of its radius (Fig 1). A 2-down, 1-up staircase procedure, ending after six reversals, was used to estimate the threshold for detecting distortion as the value with a 75% correct response rate. The threshold was recorded as a logMAR value. The hRSD test was performed on both eyes uniocularly with an optical correction for near (4m prescription plus near addition). The right eye was always tested first. Participants held the iPod at a comfortable distance and indicated the distorted shape by touching it; they were instructed to guess when unsure. The final hRSD score was the average of two consecutive tests or three tests when there was a substantial difference between the first two[24].

OCT (Heidelberg Spectralis) scans of SEs were assessed by an ophthalmologist at each visit. The assessing ophthalmologist was always masked with respect to the hRSD result. Diagnosis of nAMD was based on OCT, VA and dilated slit-lamp assessment, and in all cases was confirmed with FFA. On confirmation, anti-VEGF treatment was initiated. Participants who developed nAMD in the SE (“converters”) exited the study at this point (the “conversion visit”). Other participants (“non-converters”) exited the study having completed up to twelve study visits. For most purposes the hRSD threshold at their final visit was used in analysis.

Two experienced ophthalmologists (authors HH and SPH), masked to the results of the hRSD test, independently reviewed the OCTs and FFAs of all the converters to confirm the diagnosis. OCTs from visits prior to the clinical conversion visit were reviewed to confirm that no signs of nAMD were present prior to the date of the clinical diagnosis of nAMD. The OCT features used to define nAMD were diffuse thickening of the retina, intra-retinal cysts, subretinal fluid and subretinal hyper reflective material. Serous PED was not considered evidence of nAMD. To confirm absence of nAMD development in the SE of non-converters a two-stage grading was carried out. The OCTs of all non-converters were reviewed by a single grader (one of the authors, NPV); this identified twelve additional patients in whom OCT changes gave rise to a suspicion of conversion. These twelve plus a random sample of a further 24 non-converters (15%) were again subjected to detailed review by the ophthalmologists. In cases of disagreement, a consensus session was organised for adjudication.

### Analysis

A review of the literature suggested that we could expect approximately 10% of our participants to develop nAMD in the SE if we were able to follow them over twelve months. On this basis we recruited a sample of 202 patients to allow us to calculate test sensitivity with 95% CI’s of ±20%. Statistical analysis was conducted using SPSS (version 22; IBM Corp., Armonk, NY). Data are summarised in the text using the mean and standard deviation (SD) or 95% confidence intervals (95% CI). Analysis of variance (ANOVA) was used for multiple comparisons of group parameters; p<0.05 was taken to represent a statistically significant result. The receiver operating characteristics (ROC) curve was constructed in order to asses test performance with the aim of establishing the sensitivity and specificity of the hRSD test for detecting nAMD relative to clinical diagnosis with FFA confirmation.

## Results

**Fig 2:**
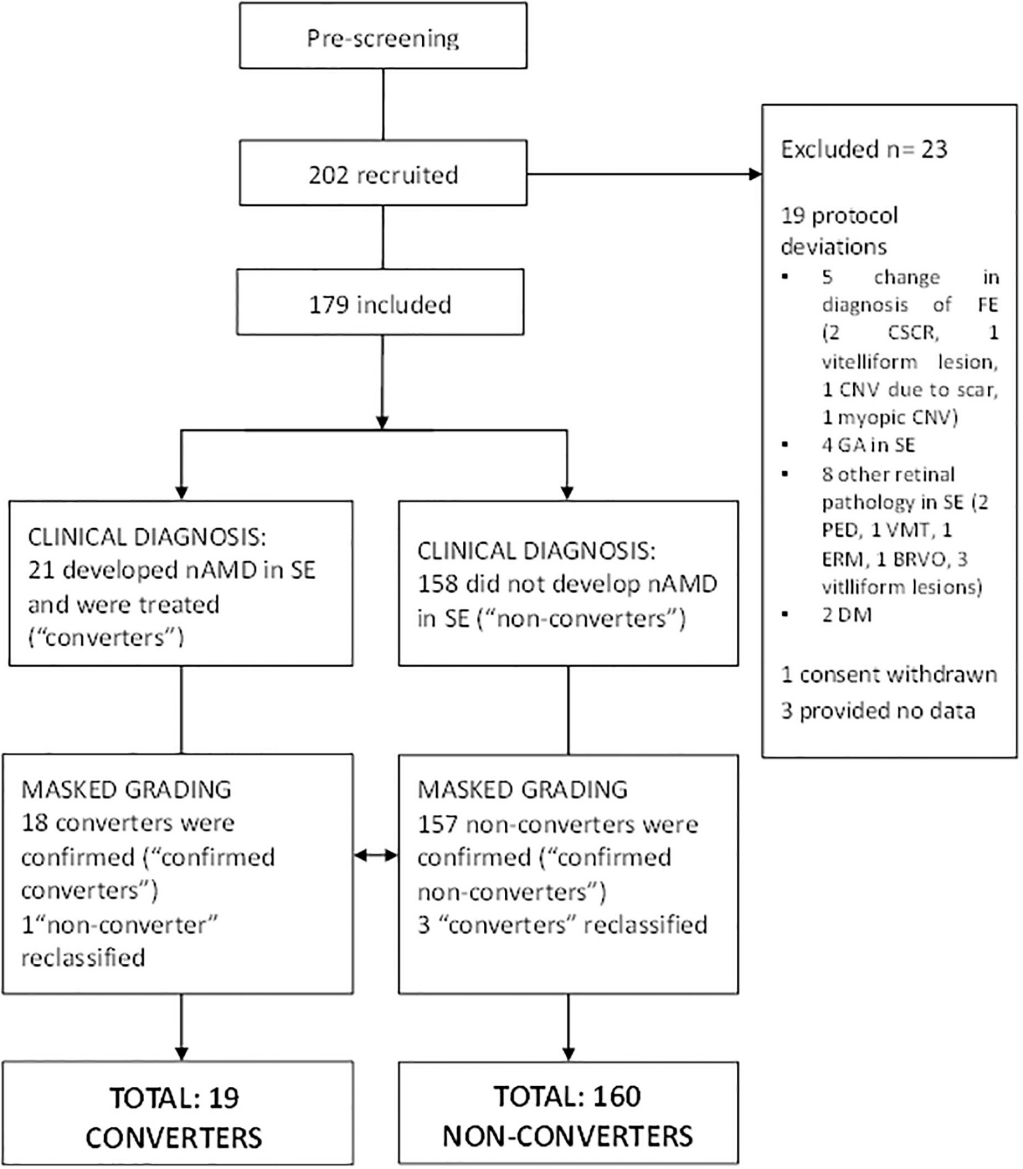
Diagram demonstrating the flow of study participants. SE: Study eye—no nAMD at recruitment; FE: First eye; diagnosis of nAMD, receiving regular assessment and treatment. CSCR: central serous chorioretinopathy; CNV; choroidal neovascularisation; GA: geographic atrophy; PED: pigment epithelium detachment; VMT: vitreomacular traction; ERM: epiretinal membrane; BRVO: branch retinal vein occlusion; DM: diabetes mellitus.

Fig 2 illustrates the flow of patients through the study. Of 202 patients recruited, 23 were excluded from analysis. Of these, 19 were classed as protocol deviations, consisting of patients found not to meet the inclusion/exclusion criteria on post-recruitment review due to undocumented lesions in the SE (e.g. geographic atrophy, pigment epithelium detachment, vitreo-macular traction, epiretinal membrane), or in whom the diagnosis in the FE changed (e.g. from nAMD to chronic serous chorioretinopathy, vitelliform lesions, scar, myopic CNV). One further patient withdrew from the study and withdrew consent for data collected to be retained, and no data were collected from three who were diagnosed with other serious illnesses or died prior to data collection commencing. Of the 179 patients (mean age 78±8y; range 52-93y; 107 female, 60%) who contributed data to the study, 77 completed twelve visits and 130 completed at least six visits. Nineteen withdrew having completed fewer than twelve study visits (but permitted us to retain their data) and thirty two were lost to follow up because they were discharged from the AMD service or stopped attending appointments. At baseline, the mean (±SD) VA, hRSD threshold and central subfield thickness (CST) for the study eye (SE) in the final group of 179 patients were 0.06±0.12logMAR, -0.53±0.22 logMAR and 276±25μm respectively.

During the study, 21 patients were identified as having developed nAMD in the SE and listed for treatment by their clinicians. Masked grading confirmed the development of nAMD in 18/21, with three reclassified as non-converters. Of the 158 non-converters, one was reclassified on masked grading. In five of the converters, OCT/FFA review indicated the development of nAMD prior to the clinical diagnosis (one visit before in all cases); the hRSD results from the earlier visit were used as the conversion value. In summary, analysis was conducted on 19 confirmed converters and 160 confirmed non-converters.

**Fig 3:**
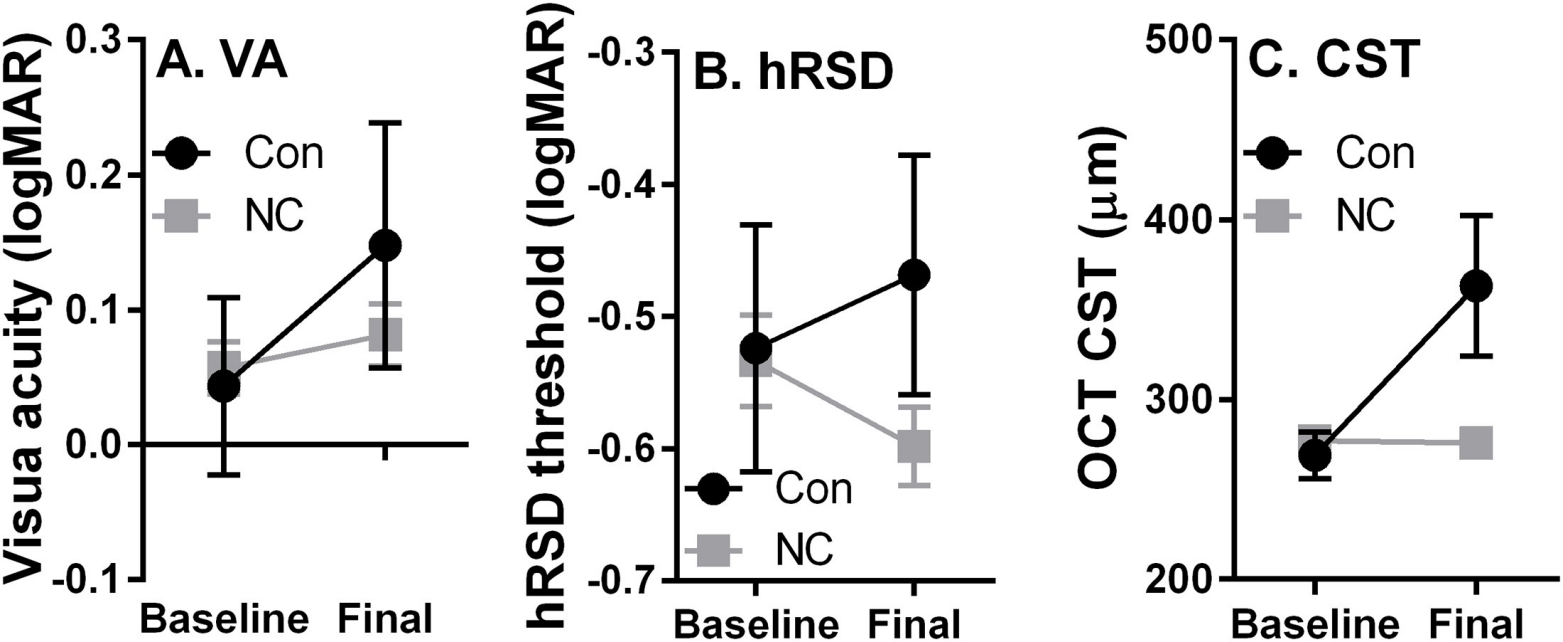
Mean (±95% CI) baseline and final VA (A), hRSD (B) results and central subfield thickness (CST; C) for 19 converters (Con) and 160 non-converters (NC). Note that in C the error bars for the non-converter data are smaller than the symbols.

At baseline, converters and non-converters were indistinguishable in terms of VA (0.05±0.14 logMAR and 0.06±0.12 logMAR respectively; mean±SD), hRSD threshold (-0.52±0.19 logMAR and -0.53±0.22 logMAR) and CST (269±26μm and 277±25 μm). The mean ages of converters and non-converters were 80±7 years and 78±7 years, respectively; these ages were not statistically different (t = 1.3; p = 0.2). We compared VA, hRSD thresholds and CST at baseline and at the conversion visit for converters and final study visit for the non-converters (Fig 3). Note that the number of visits, and therefore the time between baseline and conversion/final visits, varied both within and between groups. VA declined slightly in both converters and non-converters (Fig 3A). When tested with a repeated measures ANOVA with group (converter vs non-converters) as a between and timepoint (baseline vs final) as a within-subjects factor, timepoint returned a statistically significant main effect (F_1,174_ = 11, p = 0.001), group did not (F_1,174_ = 0.9, p = 0.375) and the timepoint x group interaction was statistically significant (F_1,174_ = 4.6, p<0.03). For hRSD threshold, there was a divergence between groups; it improved slightly in the non-converters, while it worsened in converters (Fig 3B). When tested with the same design of ANOVA as used for VA, neither timepoint nor group returned statistically significant main effects (F_1,177_ = 0.03, p = 0.87 and F_1,177_ = 2.6, p = 0.11 respectively); the timepoint x group interaction was statistically significant (F_1,184_ = 5.7, p = 0.018). For CST (Fig 3C) both timepoint and group had a statistically significant effect (F_1,170_ = 100, p<0.001 and F_1,170_ = 31, p<0.001 respectively) and the interaction was also statistically significant (F_1,170_ = 110, p<0.001).

**Fig 4:**
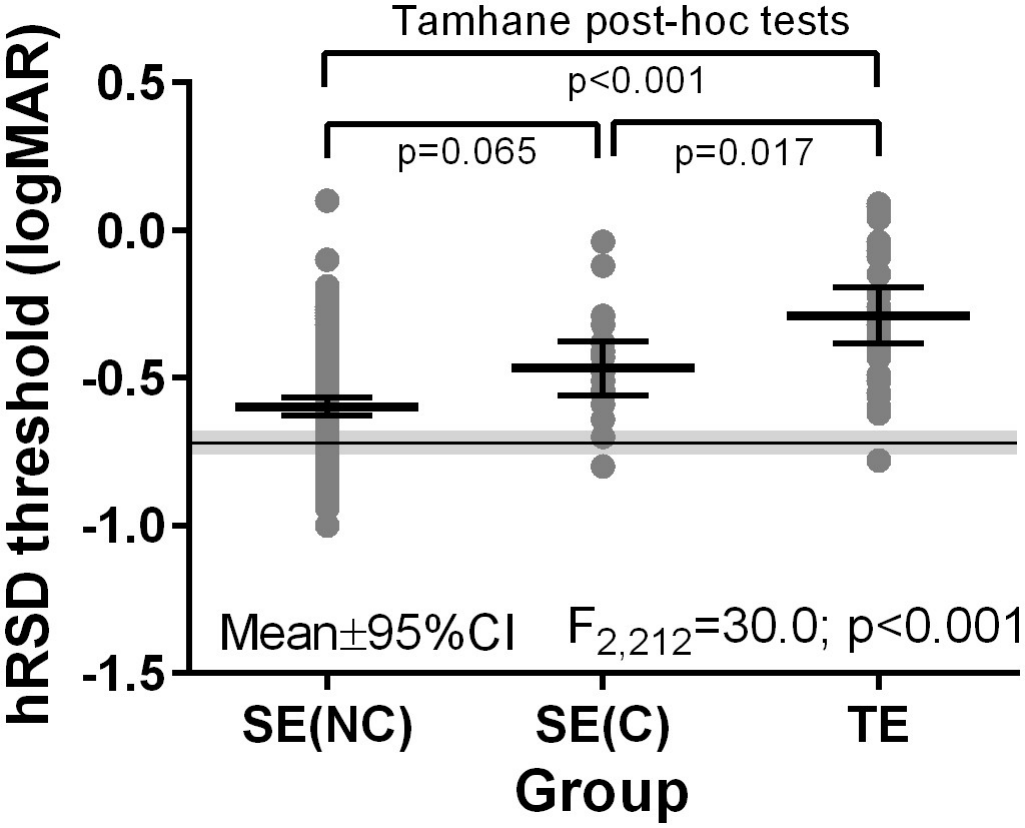
hRSD results for the study eyes in the non-converters [SE(NC); final visit], the study eyes in the converters [SE(C); conversion visit] and eyes in which nAMD has been diagnosed tested around the time of the first antiVEGF injection (FE). Datapoints indicate individual test results, the mean (±95% CI) also shown for each group. The grey horizontal line and region is the mean (±95% CI) hRSD result for healthy eyes, taken from Ku et al (2016). The one-way ANOVA result (comparing the three patient groups) and post-hoc test results (Tamhane’s T2) are shown for these data.

We identified 29 patients whose first eye (FE; the eye in which nAMD had been diagnosed), had been tested with the hRSD test around the time of their first anti-VEGF injection. Five were newly diagnosed and tested prior to their first injection, and 24 were tested no more than one month after their first injection. The FE and SE hRSD thresholds were not correlated in these patients. We take these thresholds to represent the level of function in patients as they enter treatment. The mean FE hRSD threshold was -0.29±0.25logMAR, markedly worse than the SE hRSD threshold at conversion (-0.48±0.19logMAR) or the final hRSD threshold in non-converters (-0.60±0.19; Fig 4). A one-way ANOVA of these data returned a statistically significant result (F_2,212_ = 30; p<0.001). As Levene’s test for the homogeneity of variances returned a statistically significant result (p = 0.04) for these data, Tamhane’s T2 post-hoc tests were used to further investigate the group differences. This indicated statistically significant differences between the FE and both SE results (SE non-converters p<0.001; SE converters p = 0.017); the difference between the SE of converters and non-converters did not reach statistical significance (p = 0.065). For comparison, we also extracted data for a group of healthy eyes we have previously published[18]. The mean hRSD threshold calculated for one randomly selected eye from fifty-six healthy participants (mean age 63±11y), tested with the same 3AFC version of hRSD test as used in the present study, was -0.71±0.16logMAR. We re-ran the ANOVA, comparing healthy, FE and SE thresholds (F_3,363_ = 35; p<0.001). Post-hoc testing indicated that the threshold for these healthy participants was statistically significantly different from the threshold for the SE of the non-converters as well as the other two groups (all p<0.001).

**Fig 5:**
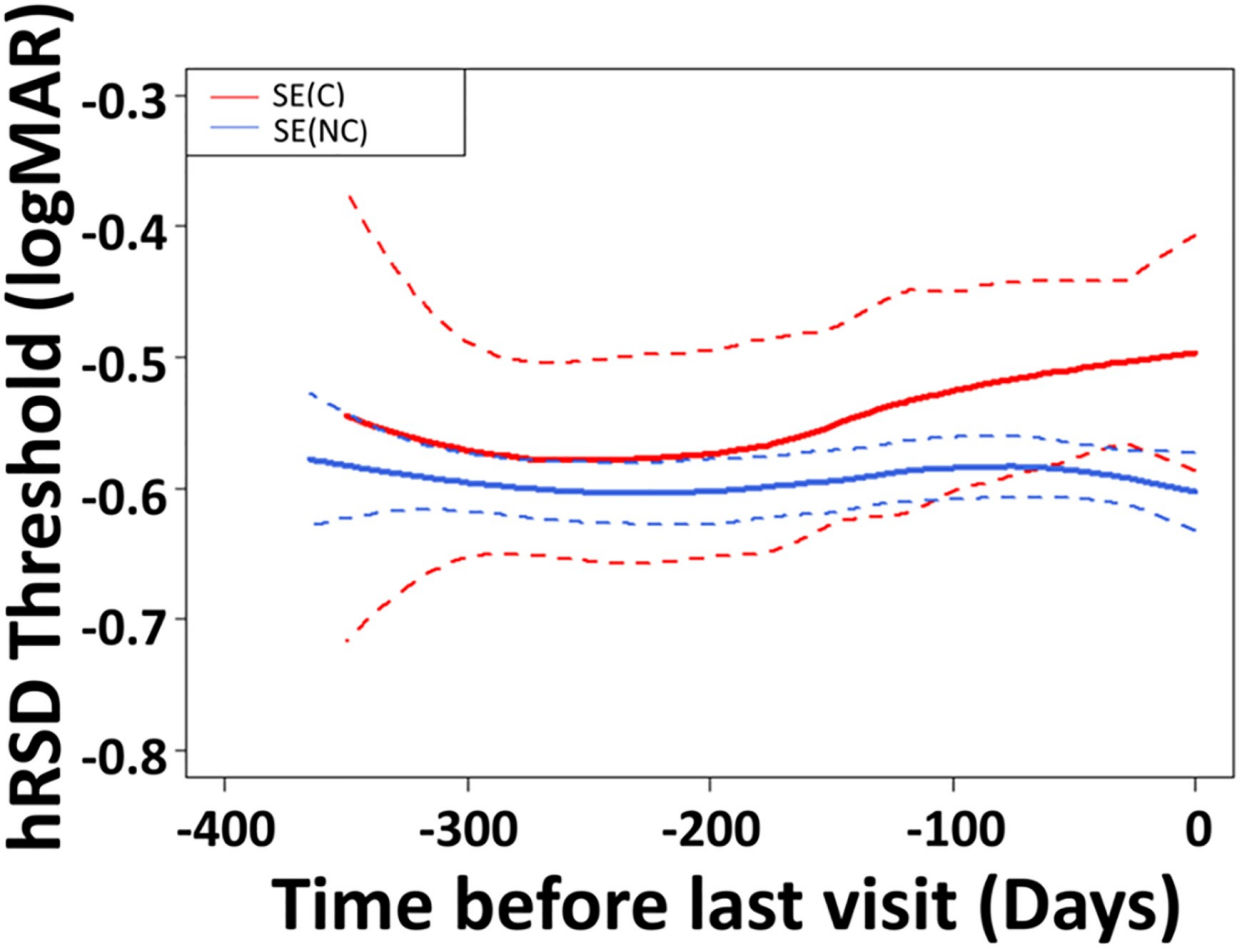
Timecourse of hRSD performance in the SE of converters (Red) and the SE in the non-converters (Blue). Solid lines show the Loess fit (±95% CI) calculated by the *loess* package in R, using a default span parameter of 0.75. The fit was aligned on the conversion or final study visit, working back in time. All available data were used.

Fig 5 illustrates the timecourse of hRSD performance in converters and non-converters by working backwards in time from the conversion visit or final study visit respectively, and fitting all the available data using a non-parametric Loess fit. This analysis implied that visual function began to decline in the converters approximately 190 days prior to the time at which it was detected clinically, and declined at an average rate of approximately 0.01 logMAR per month. Assuming this rate was maintained as disease developed, it would take a further 476 days for hRSD threshold to decline to the FE value of -0.29±0.23logMAR.

**Fig 6:**
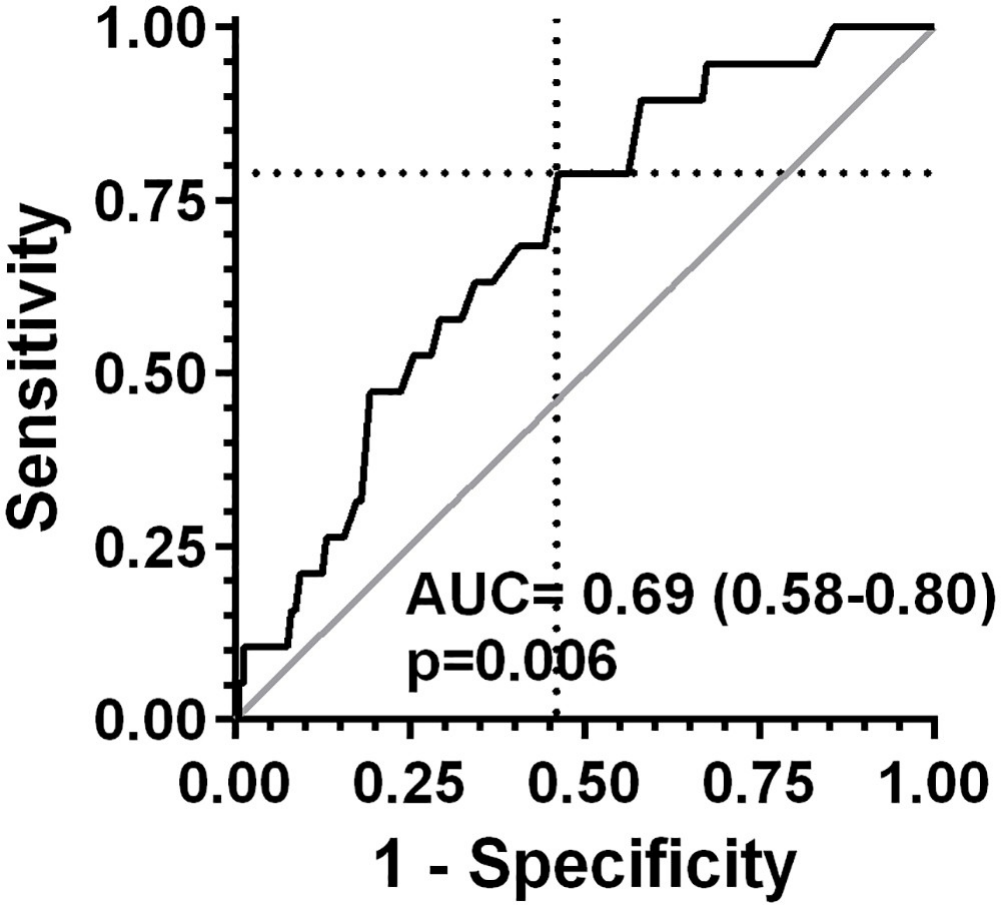
Receiver operating characteristics (ROC) curve for the performance of the hRSD test in distinguishing between converters and non-converters (ie detecting the earliest stage of nAMD). The area under the curve (AUC; 95% confidence interval) and accompanying p value are shown. Dotted lines indicate a sensitivity of 0.79 and a specificity of 0.54 at an hRSD threshold of -0.60 logMAR.

To address our primary aim of investigating the diagnostic performance of the hRSD test in distinguishing converters from non-converters, we constructed the ROC curve (Fig 6). The area under the curve (AUC) was 0.69 (95% CI 0.58–0.80), significantly different from the chance level of 0.5 (p = 0.006). The implied optimum hRSD threshold (indicated by the dotted lines in Fig 6) was -0.60 logMAR; at this threshold, sensitivity was 0.79 (0.54–0.94) with a specificity of 0.54 (0.46–0.62). The positive and negative predictive values for the cut-off threshold of -0.60 logMAR were 0.16 and 0.96 respectively. Table 2 shows the effect of varying the cut-off value for the hRSD threshold from -0.70 logMAR to -0.40 logMAR in terms of true and false positive and true and false negative results for the current data, with the accompanying negative and positive predictive values.

**Table 2:** Effect of varying hRSD cut-offs.

hRSD Cut-off (logMAR)	TP N (%)	FP N (%)	TN N (%)	FN N (%)	PPV	NPV
-0.40	5 (2.8)	25 (14.0)	135 (75.4)	14 (17.8)	0.17	0.91
-0.50	10 (5.6)	45 (25.1)	115 (65.2)	9 (5.0)	0.18	0.93
-0.60	15 (8.4)	76 (42.4)	84 (46.9)	4 (2.2)	0.16	0.95
-0.70	18 (10.1)	108 (60.3)	52 (29.1)	1 (0.56)	0.14	0.98

Relative performance of the hRSD test for a range of cut-off thresholds (from -0.40 logMAR to -0.70 logMAR). TP: True positive; FP: False positive; TN: True negative; FN: False negative; figures given as N and the percentage of the total sample of our 179 participants categorised. PPV and NPV: positive and negative predictive values respectively. The study prevalence of nAMD was 10.6%.

## Discussion

It has been shown previously that hRSD test results are stable and repeatable in healthy eyes[18], are affected by the development of macular disease, and are related to disease severity[17]. We used a prospective, longitudinal, observational study design similar to that of Do et al (2012)[22] and followed the disease-free eyes of patients being treated for unilateral nAMD. Our results confirm that, at the earliest stages of clinically detectable disease, hRSD test results are worse in eyes that develop nAMD compared to those that do not. A cross-sectional ROC analysis indicated moderate test performance in detecting the development of the earliest stages of nAMD compared to the reference standard (OCT and slit-lamp biomicroscopy with FFA confirmation).

It is difficult to directly compare our hRSD results with those published previously because of differences in patient selection and study design. Wang et al (2013)[17] reported a mean hRSD threshold of -0.36 (95% CI -0.24 to -0.48) logMAR for 11 eyes with intermediate AMD (defined as presence of large drusen and/or pigmentary changes). The mean (±SD) VA in these patients was 0.23±0.2 logMAR. This hRSD result is worse than the SE baseline hRSD threshold in our 160 non-converters (-0.53 [95% CI -0.50 to -0.57] logMAR). However, we recruited study participants on the basis of no nAMD (or other macular pathology) in the SE, and did not specify a particular level of intermediate AMD. This, along with a much better VA of 0.08±0.15 logMAR in our non-converters, suggests fundamental differences between the groups. Our non-converters’ hRSD performance was significantly worse than that of healthy participants (mean -0.71 logMAR; see the ANOVA result above and Fig 4) consistent with a decline in hRSD performance prior to the development of nAMD.

Wang et al (2013)[17] also reported a mean hRSD threshold in a group of 16 patients with “advanced AMD” (defined as either GA or exudative disease) as -0.13 (95% CI -0.02 to -0.19) logMAR. Given the mixed nature of this group, including some who had received anti-VEGF therapy, it is not directly comparable to the converters in our study (hRSD threshold at conversion -0.47[95% CI -0.38 to -0.55] logMAR). And it is also worse than that observed in 29 FE’s in our patients around the time of their first anti-VEGF injection (-0.29 [95% CI -0.20 to -0.38] logMAR).

We were able to construct the time course of hRSD threshold change in converters and non-converters, working back from the point of diagnosis or the final threshold measured respectively, (Fig 5). While these data should be interpreted with care, they do suggest functional change prior to the evidence of structural change in the central retina detected by OCT which prompts diagnosis. This is subtle and is observed in the average performance of the groups, rather than in individual patients. What is surprising is the relatively long period over which it occurs. Because converters left the study at the conversion visit and embarked on treatment, we do not know the rate of change in hRSD threshold as nAMD progresses in the absence of treatment. But these data highlight that there may be a relatively wide time window within which detection and intervention could be improved relative to current practice. If hRSD threshold were to decline at the same rate after conversion, and if we assume that the FE hRSD threshold (-0.29 logMAR) is representative of patients currently embarking on treatment, then this implies a period of 476 days between conversion and treatment. However, it seems more plausible that function might decline more rapidly post conversion, with the accumulation of fluid and structural disruption that might be expected, prompting testing and diagnosis in a shorter period than this implies. A recent review concluded that in the transition from intermediate AMD to diagnosed nAMD, VA can decline by 3–5 lines, with many patients having nAMD for 6–12 months before treatment is initiated[11]. In this general context the hRSD test could have value in prompting earlier diagnosis and treatment.

We found diagnostic performance of the hRSD test, in detecting the earliest stages of nAMD, to be moderate against the reference standard of spectral-domain OCT, slit lamp biomicroscopy and FFA. There are alternative tests, which, like the hRSD test, could be deployed away from busy assessment and treatment clinics to monitor macular vision, particularly in at-risk eyes[25]. Perhaps most familiar is the Amsler Grid test[26], which is widely used. The ForeseeHome Preferential Hyperacuity Perimeter (PHP; Notal Vision Ltd, Tel Aviv, Israel) tests alterations to Vernier hyperacuity (similar in principle to testing radial shape discrimination[27]) and has also been approved by the FDA in the US for monitoring macular vision. In small studies it has been claimed to have good performance in detecting the occurrence of nAMD in at-risk eyes[28]. It has also been demonstrated that use of the ForeseeHome device in home monitoring prompts earlier treatment as the development of choroidal neovascularisation (CNV) is detected earlier compared to normal care [29].

Faes et al (2014)[30] conducted a metanalysis of studies examining both the Amsler grid and PHP for the detection of nAMD. The pooled sensitivity of studies assessing the Amsler grid was 0.78 (95% CI 0.64–0.87), and the pooled specificity 0.97 (95% CI 0.91–0.99). For PHP they reported a pooled sensitivity of 0.85 (95% CI 0.80–0.89), and specificity of 0.87(95% CI 0.82–0.91). However, as they pointed out, many of the studies included in the analysis were small case-control series. These tend to exclude difficult to diagnose cases and therefore artificially boost the apparent sensitivity and specificity[31]. This tendency is further exacerbated by the inclusion of studies in which healthy participants are compared to patients with established disease. The apparent level of diagnostic performance implied by the high sensitivities and specificities generated by Faes et al. (2014)[30] is not likely to be achieved in clinical practice.

Importantly Do et al. (2012)[22] followed the unaffected fellow eye of patients with unilateral nAMD (the same study design which we adopted) and investigated the performance of time domain OCT, PHP and supervised Amsler grid in detecting nAMD, presenting data from a group of 87 patients who had a baseline median age of 79y and identical baseline VA and similar gender balance to our sample. Of their 87 patients, 13 (15%) converted to nAMD in the study eye. This study design allows for a more clinically relevant assessment, particularly for early detection of nAMD. At the outset patients cannot be classified into the required diagnostic categories (i.e. nAMD vs non-nAMD) so there is no scope for the “diagnostic bias” that might be present in case-control designs. The discrimination to be made is also closer to that being made clinically i.e. between intermediate AMD and new-onset nAMD, not between healthy eyes and those with established disease. Not surprisingly the sensitivities for both Amsler grid and PHP were markedly lower than those reported by Faes et al (2014)[30] at 0.42 (95% CI 0.15–0.72), and 0.50 (95% CI 0.23–0.77) respectively[22]. These results provide a valid comparison with our data given the similarity in patients recruited and study design. On this basis, the performance of the hRSD test in our study (sensitivity: 0.79 [95% CI 0.54–0.94]; specificity of 0.54 [95% CI 0.46–0.62] at a threshold of -0.60logMAR) compares favourably with these alternatives.

Caution should be exercised in comparing the performance of the hRSD test we have reported using this prospective study design, and the performance of other tests which have followed alternative designs, given the potential for bias noted above. Our aim was to investigate a single test (ie the hRSD) compared to a widely recognised clinical reference standard. It was also to assess the performance of the hRSD for detecting nAMD at an early stage, around the time it can be diagnosed (hence the longitudinal nature of the study). Clearly there would be value in comparing a number of tests to the same reference standard in a single study with the same prospective design.

Given the moderate sensitivity and low specificity of the hRSD test, the manner in which it might be deployed must be carefully considered. The comparative values for the positive and negative predictive values (PPV and NPV; Table 2) suggest a role in ruling out disease and providing reassurance to patients away from clinic settings. However, our study prevalence of nAMD (10.6%) is obviously much higher than its population prevalence. Population prevalence is influenced by many factors including age and ethnic origin[1]. For a Caucasian population aged 70-80y a prevalence of approximately 2% is realistic[1, 32]. Using the sensitivity and specificity of the hRSD test for a cut-off value of -0.60 logMAR, and assuming a prevalence for nAMD of 2%, yields a PPV of 0.03 and NPV of 0.99. However, as Table 2 illustrates, a cut-off of -0.60 logMAR is likely to generate a high number of false positive results. These can be reduced by using a more positive cut-off without materially changing the PPV and NPV. Using a cut-off of -0.50 logMAR (essentially sacrificing sensitivity) reduces false positives by half; at a prevalence of 2% the PPV and NPV are 0.04 and 0.99. Increasing the cut-off and reducing sensitivity has the inevitable effect of also increasing false negatives. However, in the case of attempted earlier detection of nAMD, a patient with a false negative result would be no worse off than in current practice. If nAMD was present, function would continue to decline until a retest detected it or symptoms prompted assessment, diagnosis and treatment.

Our study has a number of limitations. The issue of the influence of treatment in one eye on the conversion rate in the other eye has been addressed in a retrospective analysis of data from the ANCHOR and MARINA trials; monthly ranibizumab injections were not found to lower the conversion rate in fellow eyes[33]. Analysis of data from the CATT trial found no statistically significant difference in the conversion rate in fellow eyes between ranibizumab and bevacizumab[34]. However, our aim was to capture a suitable number of patients at the earliest stages of the development of nAMD in order to assess the performance of the hRSD test. Whether first or second eyes are being monitored with the test is unlikely to affect that performance. Another limitation of our study is that other than establishing that nAMD was not present in the SE, we did not characterise what level of early or intermediate AMD was present. The SE was identified as being “at risk” because of disease in the first eye. So these results are likely to be most applicable to settings in which a heterogeneous population is being monitored.

The importance of monitoring the unaffected eye in patients being treated for unilateral nAMD has recently been highlighted using real-world data[35]. Particularly in treat-and-extend regimens, as intervals between assessments increase in length, our data suggest that the hRSD test might have a role in monitoring these eyes, particularly given that this can be accomplished remotely[24]. Our patients found the test easy to use and the instructions easy to follow, as has been reported previously[17, 36]. A further application might be in primary care settings, where the high NPV might be useful in reducing false positive referrals to hospital AMD clinics.

In the context of frequent late presentation, relatively inexpensive self-monitoring with the hRSD test of people at risk of losing vision from nAMD offers potential for preserving vision. Our findings imply an extended time window within which earlier diagnosis and treatment might be possible. Future research on the deployment of the hRSD test within clinical pathways should aim to define appropriate target populations such as the general elderly population, at risk eyes, fellow eyes of affected patients and/or eyes undergoing treatment/monitoring.
